# Preliminary pharmacokinetic investigations of hydroxylated metabolites after controlled inhalative and oral consumption of hexahydrocannabinol (HHC)

**DOI:** 10.1038/s41598-026-62767-x

**Published:** 2026-07-20

**Authors:** Lisa Höfert, Willi Schirmer, Isabelle Mösch, Benjamin Franz, Cedric Groß, Susen Becker, Sven Baumann

**Affiliations:** 1 Institute of Forensic Medicine, Medical Faculty, University of Leipzig, Leipzig, Germany; 2 Institute of Forensic Medicine, Forensic Toxicology and Chemistry, University of Bern, Bern, Switzerland; 3 Department of Chemistry, Biochemistry and Pharmaceutical Sciences, University of Bern, Bern, Switzerland

**Keywords:** Hexahydrocannabinol, Cannabinoids, Pharmacokinetics, Metabolism, Forensic toxicology, Biochemistry, Biomarkers, Chemistry, Medical research

## Abstract

The pharmacokinetics of the semisynthetic cannabinoid hexahydrocannabinol (HHC) and its 11-nor-carboxy- as well as 11-hydroxy-metabolites has already been the subject of research in various recent publications. However, further metabolism studies showed the presence of other, especially (side-chain-)hydroxylated metabolites in urine and serum. This study aimed to investigate the pharmacokinetics of these metabolites after oral (25 mg fruit gum) and inhalative (three puffs from vape) consumption, using serum and urine samples from a previous study. Serum (up to 48 h) and urine (up to five days) samples of six participants (three per consumption group) were collected at different time points and analyzed by LC-QqTOF and LC-QqLIT. Three metabolites (M1: side-chain-hydroxylated; M2: hydroxylated on alicyclic moiety; M3: 8-OH-HHC) were further investigated. The peak area ratios of analytes and internal standards were plotted against the time after consumption to obtain pharmacokinetic information. Overall, this study proved the presence of these metabolites in biological samples and provided pharmacokinetic insights for the first time. Differences between serum and urine, oral and inhalative consumption as well as inter-individual differences were observed. The investigated metabolites could partially serve as unambiguous consumption markers for HHC, as they are unlikely to be formed in vivo from corresponding Δ^9^-tetrahydrocannabinol (THC) metabolites.

**Supplementary Information:**

The online version contains supplementary material available at 10.1038/s41598-026-62767-x.

## Introduction

Hexahydrocannabinol (HHC) is a semisynthetic cannabinoid, which mimics the psychoactive effects of Δ^9^-tetrahydrocannabinol (THC) from *Cannabis sativa* L. HHC is found as an epimeric mixture of (9*R*)-HHC and (9*S*)-HHC and can be synthesized from the hydrogenation of Δ^8^-THC and Δ^9^-THC^1,2^. THC and its positional double bond isomers are regulated under the United Nations Single Convention on Narcotic Drugs from 1971^3^. Nonetheless, Δ^8^-THC and Δ^9^-THC are easily obtained from cannabidiol (CBD) after acidic catalyzed cyclization^4^.

Since 2022, HHC was used as a legal alternative to THC in countries that prohibited the usage of cannabis for recreational purposes^5–8^. It was commonly marketed in the form of e-liquids, vape cartridges, fruit gums, oils, and sprayed THC-free cannabis flowers from vending machines, CBD shops, and online shops. HHC acts as a partial agonist on CB1 and CB2 receptors and is therefore assumed to produce psychoactive effects similar to those of THC, with (9*R*)*-*HHC representing the pharmacologically active diastereomer^9,10^. This can be accompanied by potential side- and toxic effects, such as anxiety, impaired psychomotor performance or psychiatric adverse effects. Several cases of HHC intoxications have already been reported in media and in scientific literature^11,12^. Consequently, most countries have meanwhile placed HHC under their narcotic substance law.

Due to the popularity of HHC usage, many publications already investigated the metabolism, detection and quantification in forensic toxicological samples^13–20^. Additionally, a few studies already investigated the pharmacokinetics of the HHC epimers and some of its major metabolites, especially 11-hydroxy-HHC (11-OH-HHC) as well as 11-nor-9-carboxy-HHC (HHC-COOH)^21,22^.

The authors of this study also already published separate studies about the metabolism and the pharmacokinetics of HHC, 11-OH-HHC and HHC-COOH, using self-administration experiments as well as a controlled study setting^9,23^. In this regard, clear similarities were observed in the pharmacokinetics of HHC and its 11-OH and -COOH metabolites compared to THC and the corresponding metabolites, particularly concerning half-lives and concentration-time profiles after oral and inhalative consumption. However, it was recently shown that these metabolites are also formed from the metabolism of THC^24,25^. Therefore, they are not suitable as sole consumption markers for HHC. More insights into the pharmacokinetics of other metabolites are required to establish other marker substances for HHC consumption. Especially different hydroxylated and side-chain hydroxylated metabolites of HHC were already discovered in previous studies^13,16^. However, no further details about the pharmacokinetics of these metabolites are known.

Therefore, the aim of this following collaborative study between Leipzig and Bern was to investigate the pharmacokinetic profiles of additional HHC metabolites for the first time, to evaluate their potential utility as specific consumption markers for HHC, since the metabolites investigated to date do not allow for a distinction between HHC and THC consumption.

## Materials and methods

### Chemicals and reagents

Deionized water (18.2 MΩ·cm) was produced with a Milli-Q^®^ IQ 7000 system from Millipore (Billerica, MA, United States) or bought from VWR International (Darmstadt, Germany). Acetic acid was purchased from Carl Roth (Karlsruhe, Germany). Methanol (MeOH) (≥ 99.9%) was bought from Carl Roth (Karlsruhe, Germany) and VWR International (Darmstadt, Germany). Formic acid (50%, in water) was acquired from Grogg Chemie (Stettlen, Switzerland). ß-glucuronidase from Patella vulgata (aqueous solution, ≥ 85,000 units/mL, used in Leipzig) and *n*-butyl acetate (*n*-BuOAc) (≥ 99.7%) were obtained from Sigma-Aldrich (Buchs, Switzerland and Steinheim, Germany). Acetonitrile (MeCN) (≥ 99.9%) was purchased from Thermo Fisher Scientific (Reinach, Switzerland) and VWR International (Darmstadt, Germany). LC-MS grade ethyl acetate and *n*-hexane were purchased from VWR International (Darmstadt, Germany). The internal standard (ISTD) (–)-Δ^9^-*trans*-tetrahydrocannabinol-D_3_ (THC-D_3_) was purchased from Cerilliant (Round Rock, TX, United States). The reference standards (9*R*)-hexahydrocannabinol ((9*R*)-HHC), (9*S*)-hexahydrocannabinol ((9*S*)-HHC), (8*S*, 9*S*)-8-hydroxy-hexahydrocannabinol ((8*S*, 9*S*)-OH-HHC), (8*R*, 9*S*)-8-hydroxy-hexahydrocannabinol ((8*R*, 9*S*)-OH-HHC), (10*R*, 9*S*)-10-hydroxy-hexahydrocannabinol ((10*R*, 9*S*)-OH-HHC), (10*S*, 9*R*)-10-hydroxy-hexahydrocannabinol ((10*S*, 9*R*)-OH-HHC), (9*R*)-11-hydroxy-hexahydrocannabinol ((9*R*)-11-OH-HHC), (9*S*)-11-hydroxy-hexahydrocannabinol ((9*S*)-11-OH-HHC), (9*R*)-11-nor-9-carboxy-hexahydrocannabinol ((9*R*)-HHC-COOH) and (9*S*)-11-nor-9-carboxy-hexahydrocannabinol ((9*S*)-HHC-COOH) were purchased from Cayman Chemical (Biomol, Hamburg, Germany). Instant buffer I and β-glucuronidase (BGTurbo, used in Bern) from finden KURA were used, provided by Specialty Diagnostix (Passau, Germany). The internal standard (ISTD) solution consisted of 0.1 µg/mL THC-D_3_ in MeOH. A solution of MeCN in water (60%V) containing formic acid (0.1%V) was used for the sample reconstitution for liquid chromatography–quadrupole time-of-flight mass spectrometry (LC-QqTOF) and liquid chromatography–quadrupole linear ion trap mass spectrometry (LC-QqLIT) analysis in Bern.

### Consumption study

A controlled consumption study with two groups (three participants each) was conducted in Leipzig. The study was ethically approved by the ethics committee of the medical faculty of the university of Leipzig (370/23-ek) and performed in accordance with the Declaration of Helsinki. Informed consent for the conduction of the study and publication of the results was obtained from all participants. Further information about the participants are listed in Supplemental Table S1.

In the inhalative group, participants were instructed to take three deep puffs from an HHC vape. In the oral group, each participant consumed one fruit gum containing 25 mg of HHC. Both products were from Cannastra, Příbram, Czech Republic and the HHC within was an epimeric mixture of 78% (9*R*)-HHC and 22% (9*S*)-HHC. The products were tested for Δ^9^-, Δ^8^-, and Δ^10^-THC, cannabidiol (CBD), cannabinol (CBN), cannabinolic acid (CBNA), cannabichromene (CBC), cannabidivarin (CBDV), cannabigerol (CBG), HHC acetate (HHC-O), hexahydrocannabiphorol (HHCP), and hexahydrocannabihexol (HHCH) using a separate, targeted LC-MS/MS MRM method in Leipzig (LOD for all analytes < 2 ng/mL). No residual THC or other of the tested cannabinoids were detected and manufacturer’s specifications were confirmed (> 99% HHC in the vape liquid; 25 mg HHC in the fruit gum). Prior to as well as in regular intervals after consumption, serum (up to 48 h) and urine (up to 5 days) samples were collected following the sampling schedule in Supplemental Table S2. More details about the study design can be found in the previously published paper^9^.

### LC-QqTOF analysis

#### Bern

The sample pretreatment was performed according to Schirmer et al. with slight modifications to the deglucuronidation step^23^. Briefly, 200 µL urine, 50 µL ISTD, 100 µL instant buffer I and 5 µL β-glucuronidase solution (BGTurbo) were incubated at 50 °C for 60 min. 500 µL *n*-BuOAc was added, the mixture was shaken for 10 min and centrifuged for 10 min (13000 rpm (17190 × *g*), 8 °C). Afterwards, the organic phase was transferred to an autosampler vial, evaporated to dryness under a stream of nitrogen at 50 °C and reconstituted with 200 µL reconstitution solution for further LC-MS/MS analysis. A Dionex Ultimate 3000 HPLC system (Thermo Fisher Scientific, Reinach, Switzerland) coupled to a TripleTOF 5600 mass spectrometer was used (Sciex, Toronto, Canada). Analyst TF software (version 1.7) and Peak View (version 1.2.0.3) (Sciex, Toronto, Canada) were used for data acquisition and processing. Mass spectra were measured in positive ionization mode with an IonDrive Turbo V ion source with TurboIonSpray probe. The curtain gas and the ion source gases 1 and 2 were set to 55 psi, the ion spray voltage floating was 5500 V, and the source temperature was 650 °C. The collision energy at Q1 was set to 10 V and the declustering potential was set to 60 V. Chromatographic separation was performed on a Kinetex C8 column, 50 × 2.1 mm, 2.6 μm, 100 Å (Phenomenex, Basel, Switzerland). A gradient method was used consisting of mobile phase A (0.1% aqueous formic acid (%V)) and mobile phase B (MeCN with 0.1% formic acid (%V)) with the following gradient: 0–5 min: 45% B, 5–14.5 min: 45–62% B, 14.5–14.6 min: 62−45% B, 14.6–15.0 min: 45% B. The injection volume was 2.5 µL, the column oven was set to 25 °C and the flow rate was 0.3 mL/min. The LC-QqTOF instrument was operated in IDA (information dependent data acquisition) and in SWATH mode (sequential window acquisition of all theoretical mass spectra). For IDA, a survey scan from *m/z* 100 to 950 was applied which triggered the acquisition of product ion mass spectra from *m/z* 50 to 950. The accumulation time was set to 50 ms and 40 ms for the survey scan and the dependent scan, respectively. For SWATH mode, a mass range from *m/z* 100 to 950 was scanned acquiring product ion spectra in windows of 35 Da from *m/z* 50 to 950. A scan speed of 35 ms for each window was applied. A collision energy with collision energy spread of 35 ± 15 V was applied for IDA and SWATH acquisition. The declustering potential was 60 V.

### LC-QqLIT analysis

#### Bern

Reference solutions were prepared by mixing 2 µL of a 1 µg/mL solution and 20 µL ISTD. The solution was evaporated to dryness and reconstituted in 200 µL reconstitution solution. The extracts after deglucuronidation as described above were measured. A slightly changed protocol was used as described previously^23^. A Dionex Ultimate 3000 HPLC system (Thermo Fisher Scientific, Reinach, Switzerland) was used, coupled to a QTRAP 4500 mass spectrometer (Sciex, Toronto, Canada) equipped with an IonDrive Turbo V ion source with TurboIonSpray probe. The curtain gas was set to 40 psi, the ion spray voltage was 3500 V, and the source temperature was 600 °C. Ion source gas 1 was set to 40 psi and ion source gas 2 was set to 60 psi. Data were acquired and processed with Analyst TF software (version 1.7) and visualized with Sciex OS (version 2.0.0.45330) (Sciex, Toronto, Canada). The same chromatographic parameters were used as decribed in the LC-QqTOF section. The injection volume was 5 µL. Spectra were acquired in positive ionization mode with a multiple reaction monitoring method (MRM). The relevant transitions with corresponding potentials of the MRM method are shown in Table 1.

**Table 1:** MRM transitions of the LC-QqLIT method from Bern. DP: Declustering potential, CE: collision energy, CXP: cell exit potential. Transitions used for the semi-quantification are marked with an asterisk (*). A dwell time of 20 ms was used for the transitions of THC-D_3_, the dwell time for the other transitions was 35 ms. An entrance potential of 10 V for every transition was applied.

Analyte	Transition, m/z	DP, V	CE, V	CXP, V
THC-D_3_	318.2→196.3*	88	32	7
	318.2 → 123.0	100	43	8
HHC	317.2 → 193.2	88	32	7
	317.2 → 123.0	100	43	8
OH-HHC	333.2 → 315.2*	87	21	12
	333.2 → 193.2	90	35	7
	333.2 → 191.2	90	35	7
HHC-COOH	347.2 → 329.3	93	23	12
	347.2 → 301.2	102	28	12
	347.2 → 193.2	102	28	12

#### Leipzig

Sample preparation was conducted as previously described by Höfert et al., applying an acidic liquid-liquid-extraction with hexane/ethyl acetate (80/20, v/v) after hydrolysis with β-glucuronidase^9^. The analysis was utilized on an Agilent 1290 Infinity II LC system (Agilent Technologies, Waldbronn, Germany) coupled with a QTRAP 5500 mass spectrometer, controlled with the Analyst^®^ 1.7.1 software (Sciex, Darmstadt, Germany). Chromatographic separation was achieved by using a Gemini^®^ 3 μm NX-C18 110 Å 150 × 3 mm analytical column (Phenomenex, Aschaffenburg, Germany). The injection volume was 5 µL. Further instrumental details can be found in Höfert et al.^9^. The previously described scheduled multiple reaction monitoring (sMRM) method was used and expanded by the 8-OH- and 10-OH-HHC metabolites. The parameters for the new analytes are detailed in Table 2. The MRM detection window was 50 s with a target scan time of 0.5 ms per sMRM experiment.

**Table 2:** MRM transitions of the LC-QqLIT method from Leipzig DP: Declustering potential, CE: collision energy, CXP: cell exit potential. An entrance potential of 10 V for every transition was applied.

Analyte	Retention time, min	Transition, m/z	DP, V	CE, V	CXP, V
(8*S*, 9*S*)-OH-HHC	7.43	333.2 → 193.2	80	33	16
		333.2 → 123.0	80	45	16
(8*R*, 9*S*)-OH-HHC	7.51	333.2 → 193.1	80	35	12
		333.2 → 123.1	80	49	18
(10*R*, 9*S*)-OH-HHC	8.84	333.2 → 315.3	80	23	6
		333.2 → 193.1	80	35	12
(10*S*, 9*R*)-OH-HHC	11.08	333.2 → 315.3	80	25	8
		333.2 → 123.1	80	53	12

## Results and discussion

The aim of this study was first to identify unknown hydroxylated metabolites of HHC in the study samples using the LC-QqTOF as well as the LC-QqLIT analysis in Bern. Second, the samples were analyzed for different commercially available hydroxylated metabolites (8-OH- and 10-OH-HHC epimers) using the LC-QqLIT method in Leipzig. Finally, pharmacokinetics of the identified hydroxylated metabolites should be further investigated.

### Metabolite identification

**Fig. 1:**
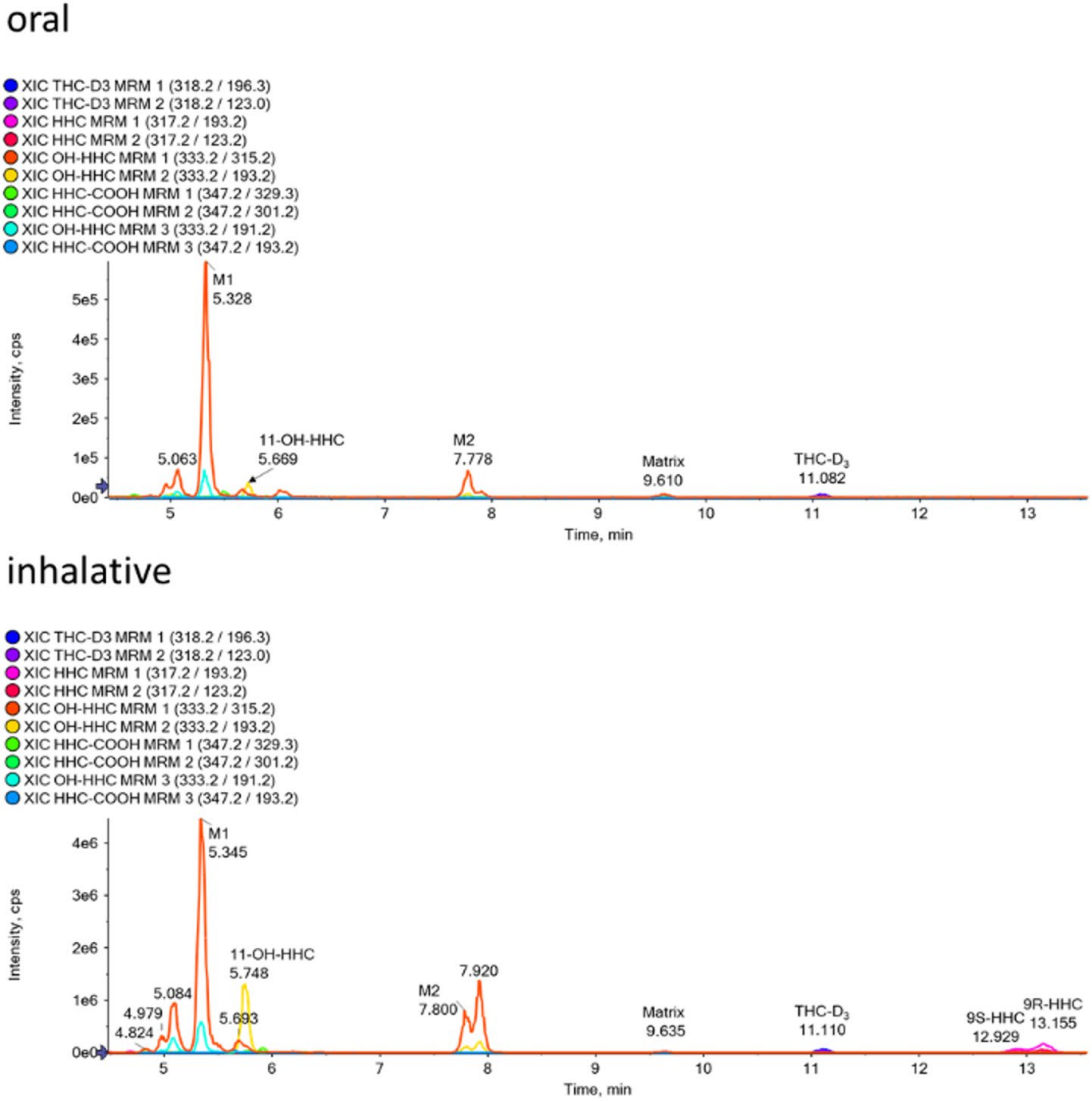
Extracted ion chromatograms of deglucuronidated urine samples from the participant O2, 2 h after the oral ingestion of HHC (top) and from the participant I2, 1 h after the inhalation of HHC (bottom) using the LC-QqLIT method in Bern.

For identification of potentially unknown metabolites, all urine and serum samples were prepared as described above and analyzed in Bern. The chromatograms of representative deglucuronidated urine samples after oral ingestion and inhalation, acquired using the LC-QqLIT method in Bern, are shown in Fig. 1.

**Fig. 2:**
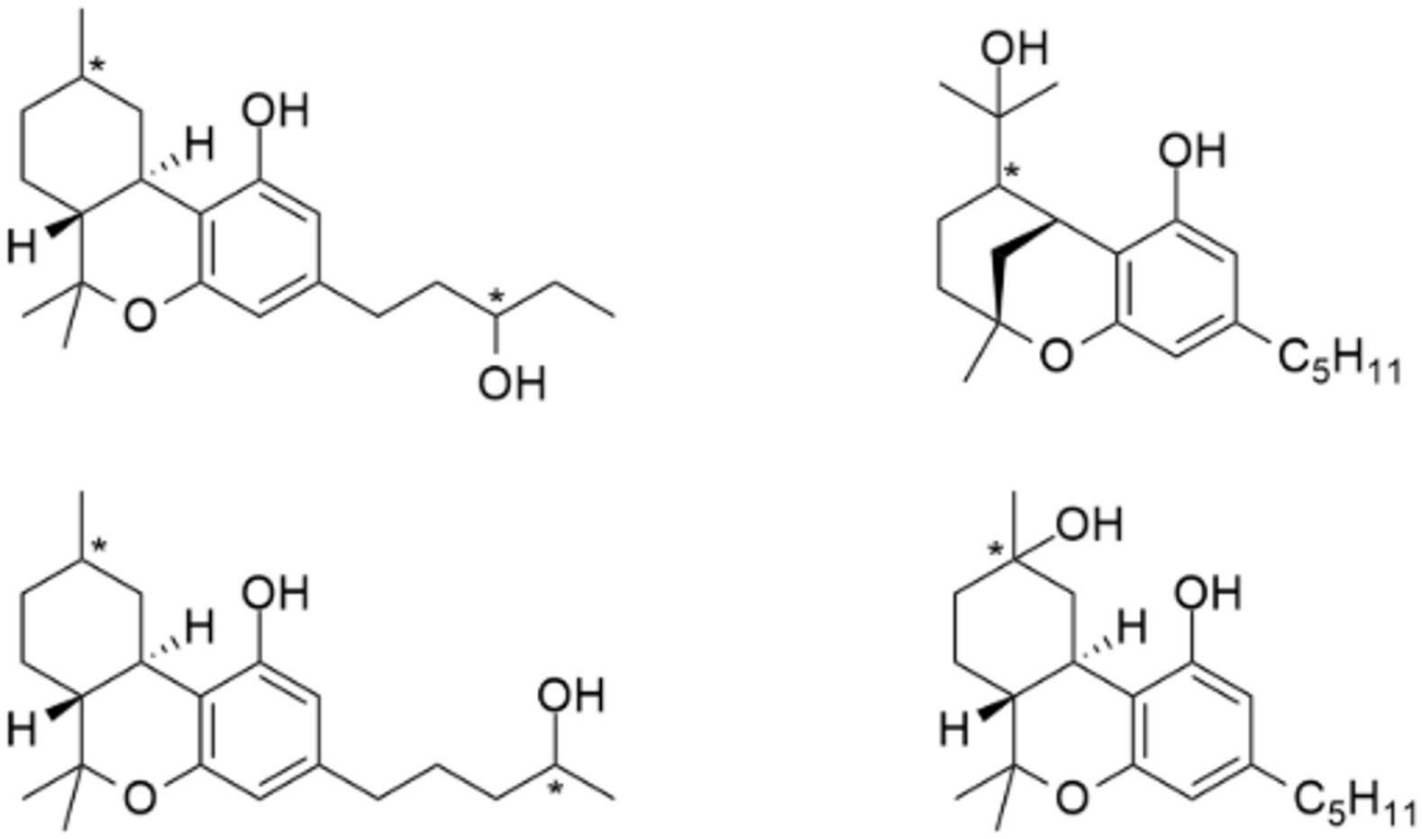
Plausible structures for the metabolites M1 (left) and M2 (right). Top left: 3’OH-HHC^15^, bottom left: 4’OH-HHC^23^, top right: 8-OH-*iso*-HHC^26^, bottom right: 9-OH-HHC^26^.

Two unknown metabolites M1 and M2 were further investigated during this research. Plausible structures are shown in Fig. 2. The metabolite M1 is a side-chain hydroxylated metabolite of HHC as it shows the mass transition *m/z* 333 → *m/z* 191, which is typical for side-chain hydroxylated metabolites of HHC^13,15,23^. This metabolite was previously tentatively identified as a diastereomer of 4’OH-HHC^23^. A urine sample 1 h after the inhalation of 20 mg HHC from this previous study was analyzed with the method described above and showed a nearly identical chromatogram as shown in Fig. 1 (bottom). Pitterl et al. identified a similar side-chain hydroxylated metabolite of HHC, 3’OH-HHC, which would also be a plausible structure for the metabolite M1^15^. The metabolite M2 is an unknown metabolite that is hydroxylated on the alicyclic moiety as it shows the presence of the fragment ion *m/z* 135 instead of *m/z* 137, similarly to the known metabolite 11-OH-HHC. An epimer of 9-OH-HHC might be a plausible structure for M2. It is however possible that this compound derives from *iso*-HHC, making 8-OH-*iso*-HHC another plausible structure^26^.

**Fig. 3:**
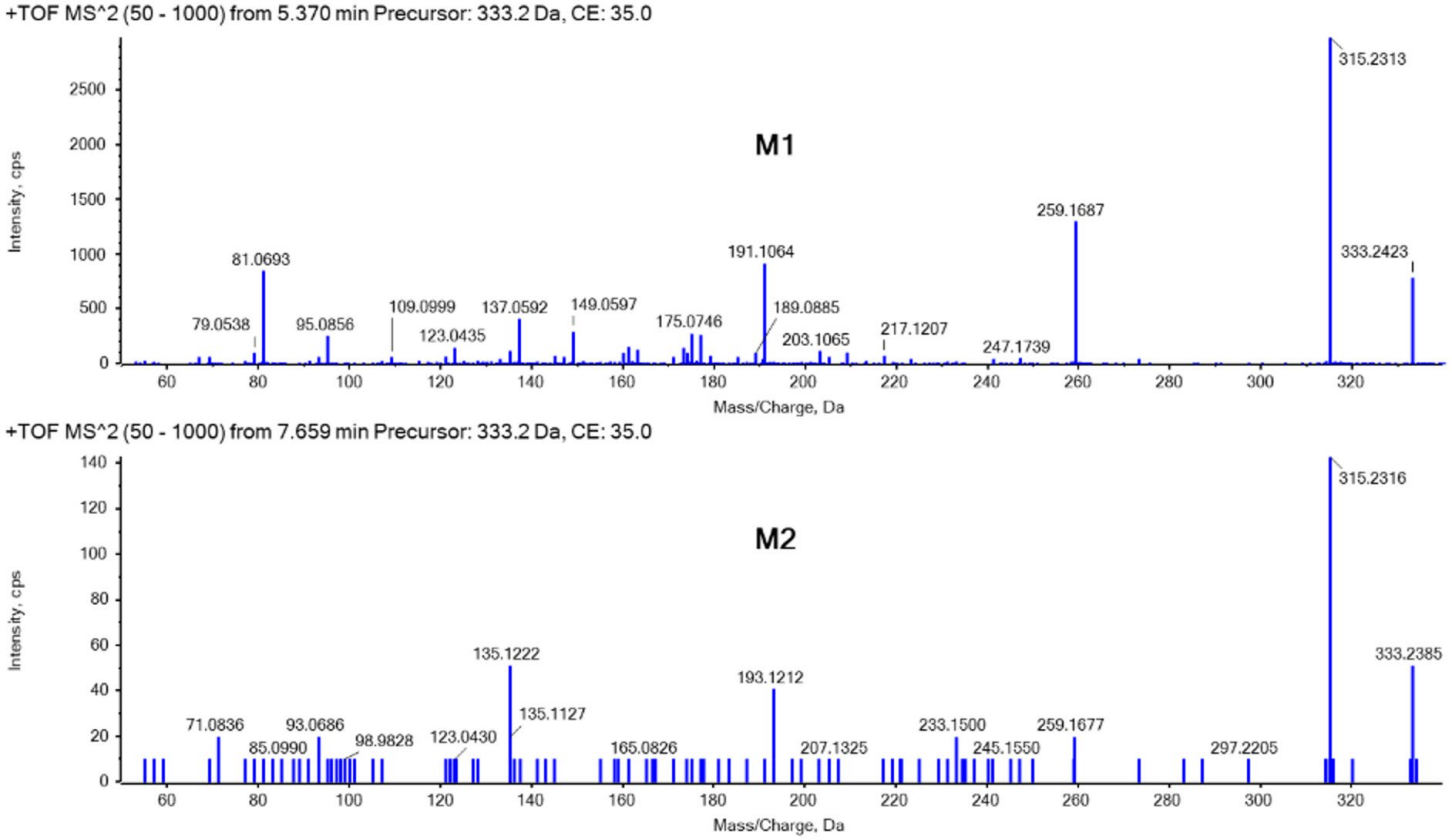
ESI+ HRMS spectra of the investigated metabolites M1 (participant O1, urine, 2 h) and M2 (participant O1, serum, 1 h) using the LC-QqTOF method in Bern.

These two metabolites were chosen as they showed similar or even higher abundance with the applied collision energies than the commonly used epimers of 11-OH-HHC. The ESI+ HRMS spectra of the metabolites M1 and M2 are shown in Fig. 3.

**Fig. 4:**
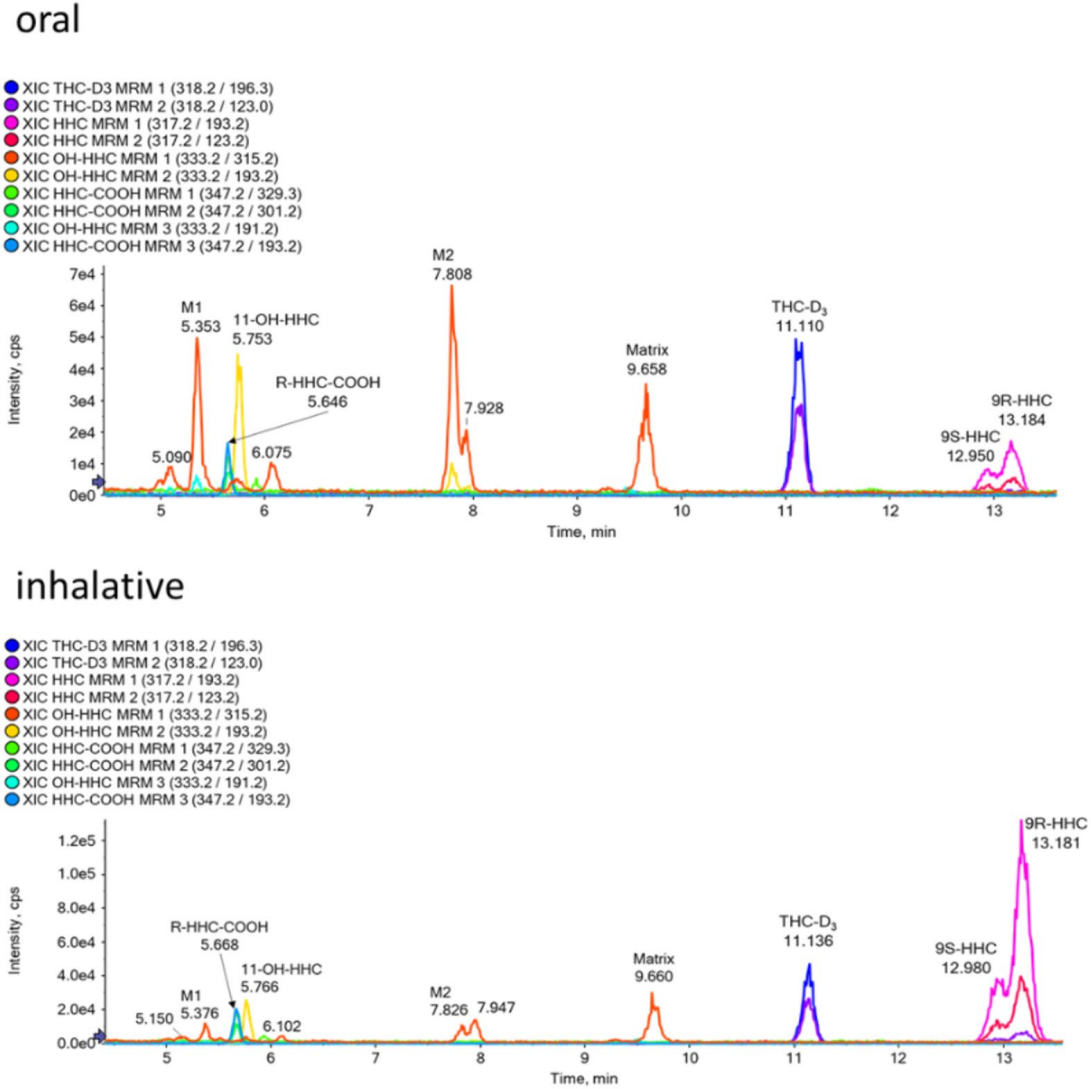
Extracted ion chromatograms of deglucuronidated serum samples from the participant O2, 50 min after the oral ingestion of HHC (top) and from the participant I2, 15 min after the inhalation of HHC (bottom) using the LC-QqLIT method in Bern.

The chromatograms of representative serum samples after oral ingestion and inhalation, acquired using the LC-QqLIT method in Bern, are shown in Fig. 4.

The same metabolites M1 and M2 have also been identified in serum samples after oral and inhalative HHC consumption. The 11-OH-HHC metabolites and the carboxy metabolites were more abundant in serum samples. The carboxy metabolites were barely detectable in the urine samples.

Overall, the first part of this follow-up study showed, that the metabolites M1 and M2 were found in urine and serum samples. In the chromatograms of serum samples after inhalative HHC consumption the metabolites 11-OH-HHC and 11-nor-(9*R*)-HHC-COOH seem to be the main metabolites. Other metabolites are of much lower abundance in all three participants. In contrast, in serum samples of the participants, which orally ingested HHC, different ratios could be observed. 11-OH-HHC and 11-nor-(9*R*)-HHC-COOH are of similar abundance as the metabolites M1 and M2 in these cases. This is possibly a consequence of the different absorption, bioavailability and distribution depending on the form of consumption.

Interestingly, the urine samples from the inhalative and oral groups show similar metabolite ratios. The most abundant metabolite in the urine samples is the metabolite M1. The epimers of 11-OH-HHC are of much lower relative abundance and the HHC-COOH epimers are barely present.

In Leipzig, the samples were analyzed for different commercially available 8-OH- and 10-OH-HHC epimers using a sensitive sMRM method. The limits of detection (LOD, S/*N* ≥ 3) were around 0.5 ng/mL and 0.1 ng/mL for the 8-OH- and 10-OH-HHC epimers, respectively. However, only 8-OH-HHC (= M3) was detectable in all urine samples except from I1, with substantial higher signal intensities after oral compared to inhalative consumption. In the serum samples, no 8-OH-HHC was detectable. Unfortunately, it was not possible to chromatographically baseline separate the 8-OH-HHC epimers, which makes it difficult to identify the epimer uniquely. However, a little difference in retention times suggests that the detected epimer is (8*S*, 9*S*)-OH-HHC. This is further supported by the findings of Di Trana et al., who detected (8*S*, 9*S*)-OH-HHC also only in urine^21^. Neither of the two examined 10-OH-HHC epimers was detectable. However, other publications already reported the presence of different 8- and 10-OH-HHC epimers in serum and urine^13,16,27^. Reasons for the absence in this study could be metabolite concentrations below the limit of detection or decomposition processes due to the long storage period of the study samples (9 to 16 months between original study and measurement for this follow-up study).

Furthermore, in all urine and serum samples from both groups except in serum from I1 a HHC isomer was found that eluted after (9*S*)-HHC and (9*R*)-HHC. This HHC isomer showed the same fragment ions as the epimers of HHC and was tentatively identified as *iso*-HHC, an indicator that the HHC within these products were synthesized from CBD^28^. 

### Pharmacokinetic investigations

In the second part of the study, the pharmacokinetics of the two unspecified metabolites M1 and M2 as well as the identified 8-OH-HHC epimer (M3) should be further investigated.

Both unspecified metabolites M1 and M2 were detected in the urine of all participants after oral and inhalative consumption. M3 was also detectable in all samples after oral consumption as well as in a few samples of I2 and I3, but was absent in the urine of participant I1. The participant I1 did not notice any psychoactive effects and showed the lowest maximum serum concentrations for 11-nor-(9*R*)-HHC-COOH, the epimers of 11-OH-HHC and HHC in the previous study^9^. This indicates that this individual incorporated less HHC and that the isomer is probably below the instrumental detection limit. This is possibly due to a less deep inhalation technique, as I1, unlike I2 and I3, was not a regular smoker (see Supplemental Table S1). This again highlights the influence of various inter-individual factors, which makes it difficult to compare concentrations, especially after inhalation.

**Fig. 5:**
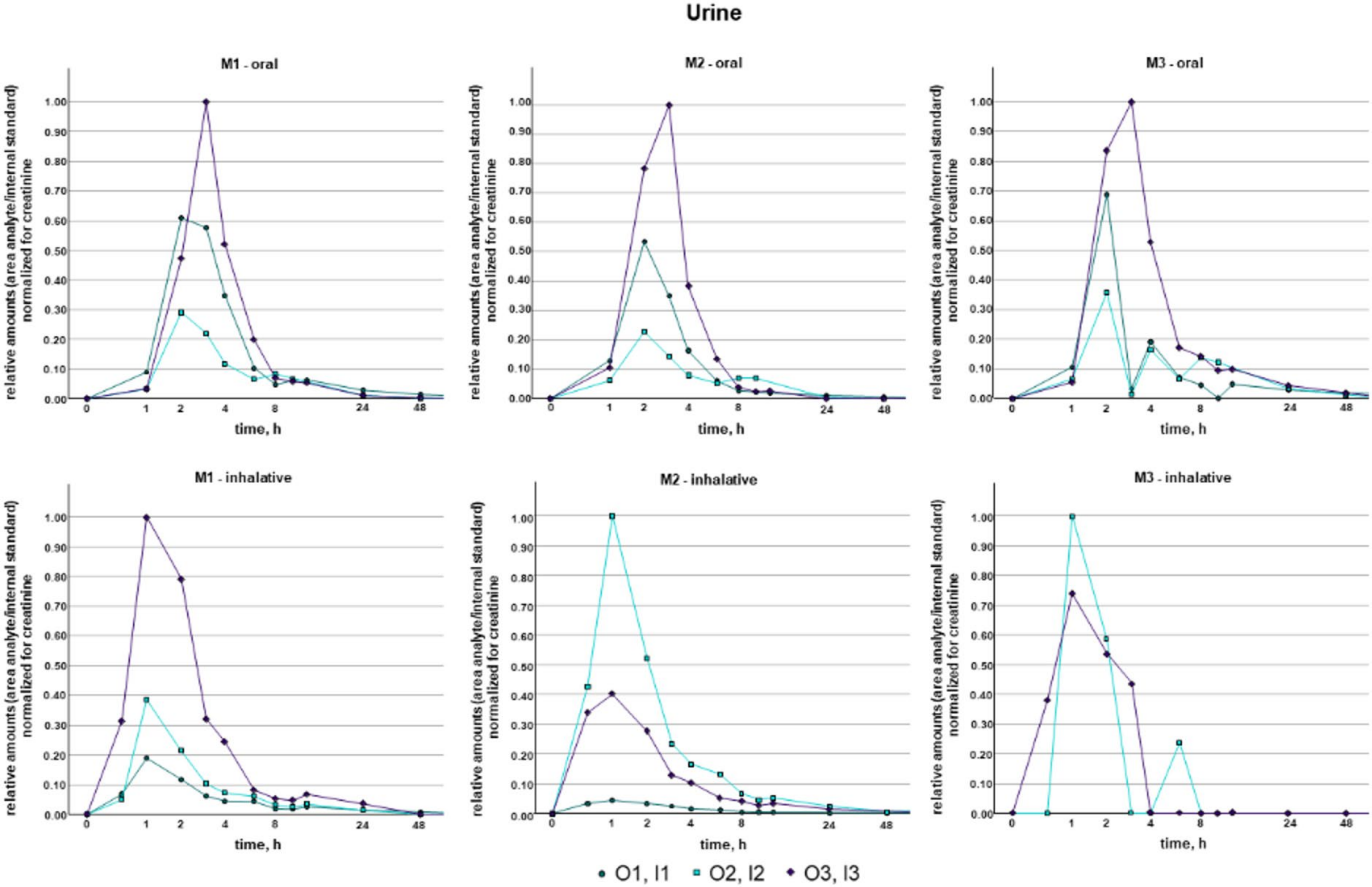
Relative amounts (ratio of peak areas of analyte and internal standard, normalized for creatinine) of M1, M2, and M3 (8-OH-HHC) in urine in relation to time after oral (top) and inhalative (bottom) consumption for each participant using the LC-QqLIT methods in Bern (M1, M2) and Leipzig (M3).

The maximum amounts of the three investigated metabolites in urine were reached 1 h after inhalative consumption and 2–3 h after oral consumption. Figure 5 shows the relative amounts (ratio of peak areas of analyte and internal standard) of the metabolites in urine normalized for creatinine and normalized to 1.

**Fig. 6:**
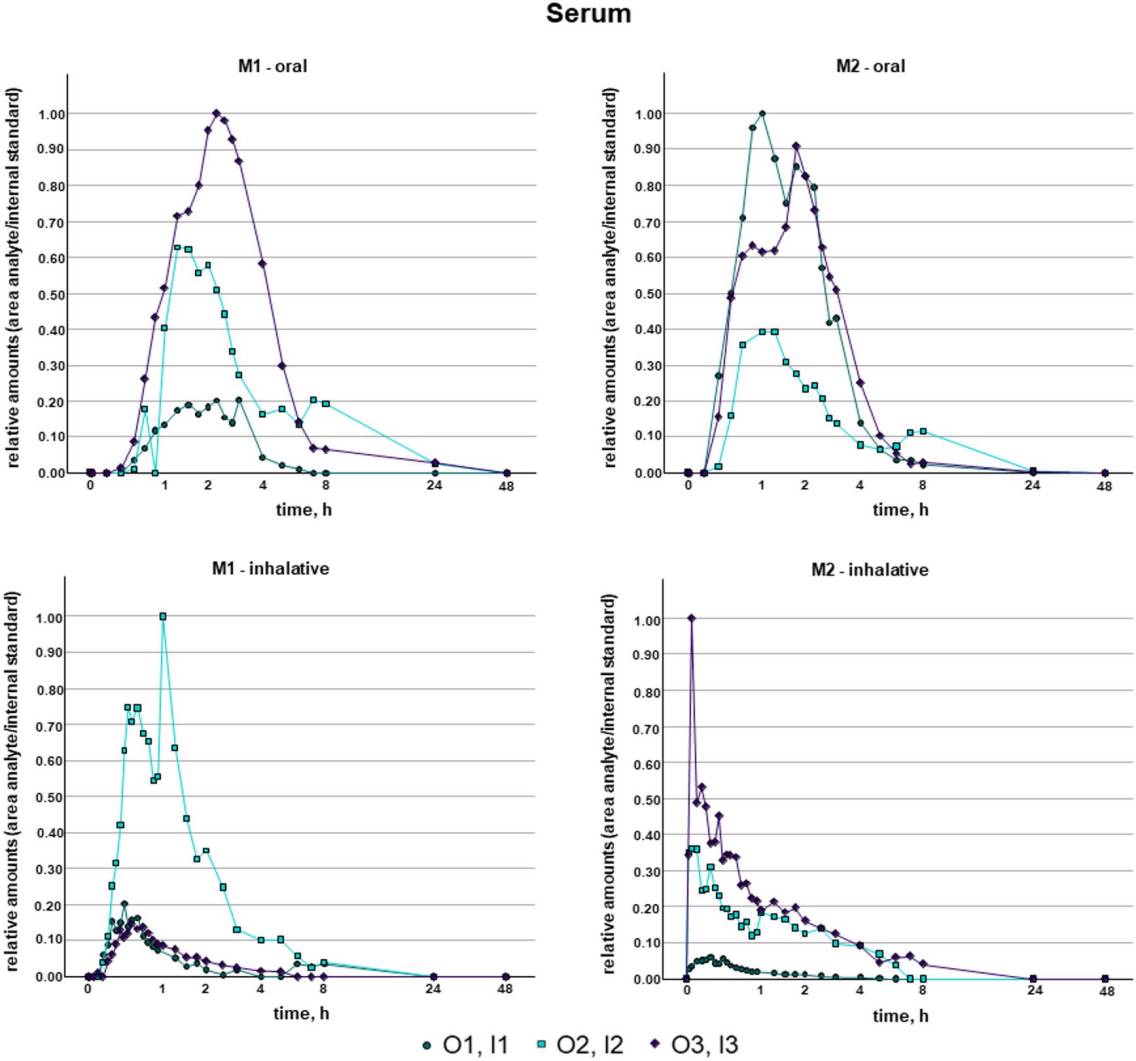
Relative amounts (ratio of peak areas of analyte and internal standard) in serum in relation to time after oral (top) and inhalative (bottom) consumption for each participant using the LC-QqLIT method in Bern.

Figure 6 shows the temporal patterns for the relative amounts in serum of M1 and M2 after oral and inhalative administration. In serum, only the metabolites M1 and M2 could be detected. Neither one of the 8-OH- nor 10-OH-HHC metabolites with available reference material were detected in serum. The maximum amounts of M1 and M2 were reached between 1 and 2.5 h after oral consumption. For M1, the maximum amount was reached between 0.5 and 1 h after inhalative consumption. In contrast, the maximum amounts of M2 were reached already within a few minutes after inhalation.

The relative abundances of the metabolites were however different between the study participants but also between the matrices from the same individuals. For example, in the inhalative group, the side-chain hydroxylated metabolite M1 was most abundant in the urine of participant I3. The same metabolite was, however, low abundant in the serum of the same individual. Additionally, the ratios between the metabolites differed, especially after inhalative consumption. For example, I2 is the person with the highest relative abundance of M1, while I3 has the highest relative abundance of M2. This indicates inter-individual differences in pharmacokinetics and metabolism.

In comparison to already investigated metabolites, it is worth noting that in serum especially M2 shows an earlier maximum than 11-OH-HHC after oral (t_max_ M2: 1–2 h; t_max_ 11-OH-HHC: 2,25 –3 h) as well as inhalative (t_max_ M2: 3–15 min; t_max_ 11-OH-HHC: 6–60 min) consumption. In urine, all three metabolites show an earlier maximum compared to 11-OH-HHC after inhalation (t_max_ M1, M2, M3: 1 h; t_max_ 11-OH-HHC: 2–4 h)^9^. This might indicate the suitability of these metabolites as possible markers for acute HHC consumption. However, further data are required, preferably with quantified concentrations, for which available reference standards of the substance would be necessary.

### Limitations

The investigated metabolites M1 and M2 were not quantified as no reference standards are available for these two compounds. Relative abundances were used for comparison at specific collision energies. Many different hydroxylated metabolites of HHC are present in the samples, which do show the same mass transitions (*m/z* 333 → *m/z* 315 and either *m/z* 333 → *m/z* 193 or *m/z* 333 → *m/z* 191) but different optimal collision energies. Additionally, different sensitivities of the instrument to the unknown metabolites contribute to their under- or overrepresentation. Additionally, for 8-OH- and 10-OH-HHC only two epimers per metabolite were investigated because of the limited availability of commercial reference material for the other epimers. Since this study is a follow-up to the original study, the samples were stored for a longer period than usual prior to analysis (up to 16 months). Although they were always stored at -80 °C and stability over this time period was at least approved for 11-OH-HHC in serum, degradation processes and associated changes in the metabolite profile cannot be ruled out with certainty. The metabolite M2 shows a similar concentration-to-time profile as the epimers of HHC that are shown in the previous study^9^. This might indicate that the compound M2 was initially present in the HHC sample, rather than being formed in vivo, which was not tested. It is therefore possible that M2 is an impurity; an epimer of 8-OH-*iso*-HHC or 9-OH-HHC would be plausible structures. Both of them can be formed from CBD under aqueous and acidic conditions as shown by Watanabe et al., the formation of the latter might also happen in vivo from the metabolism of HHC^26^. In this study no THC positive samples were tested, to verify if the identified metabolites M1-M3 might form from THC or its metabolites.

## Conclusions

This follow-up project of the already conducted study showed new insights into the metabolism of HHC as well as the pharmacokinetics of selected main hydroxylated metabolites. The investigated metabolites could serve as unambiguous consumption markers for HHC, especially for acute consumption, as they are unlikely to be formed in vivo from corresponding THC metabolites and show early peak serum concentrations. Only 11-OH-HHC and HHC-COOH have been described as THC metabolites, HHC was not found^24^. It is therefore suspected that the involved reductase requires an allylic alcohol or aldehyde to form 11-OH-HHC and HHC-COOH after the consumption of THC. The metabolite M3 might form through a similar pathway from the minor THC metabolite 8-OH-Δ^9^-THC due to being an allylic alcohol, but this was not reported yet to our knowledge. The metabolites M1 and M2 do not share these structural features and are therefore unlikely metabolites of THC. However, M2 might be an impurity in the commercial HHC product, rather than a metabolite of HHC due to its initial presence in the serum samples from the inhalative group. Emphasis should therefore be given for the presence of M1 in case samples. As long as the metabolites M1 and M2 are not unambiguously identified and available as reference standards, the ratio of HHC-COOH to THC-COOH should be used as an indicator for the consumption of HHC in real case samples as suggested by Grapp et al.^25^. 

## Supplementary Information

Below is the link to the electronic supplementary material.

Supplementary Material 1

## Data Availability

The datasets generated during and/or analyzed during the current study are available from the corresponding author on reasonable request.
