# Supplementary material for: Preliminary pharmacokinetic investigations of hydroxylated metabolites after controlled inhalative and oral consumption of hexahydrocannabinol (HHC)
Source: Sci Rep. 2026 Jul 20;16:22712. doi: 10.1038/s41598-026-62767-x (PMC13385868; doi:10.1038/s41598-026-62767-x)
Supplement: Supplementary file 1 — Supplementary Material 1 [file 41598_2026_62767_MOESM1_ESM.pdf]

Supplementary Information

Preliminary pharmacokinetic investigations of hydroxylated metabolites after controlled inhalative and oral consumption of hexahydrocannabinol (HHC)

Lisa Höfert[1, #, *], Willi Schirmer[2, 3, #], Isabelle Mösch[2], Benjamin Franz[1], Cedric Groß[1], Susen Becker[1], Sven Baumann[1]
[*] corresponding author (lisa.hoefert@medizin.uni-leipzig.de)
[#] contributed equally

[1] Institute of Forensic Medicine, Medical Faculty, University of Leipzig, Germany
[2] Institute of Forensic Medicine, Forensic Toxicology and Chemistry, University of Bern, Switzerland
[3] Department of Chemistry, Biochemistry and Pharmaceutical Sciences, University of Bern, Switzerland

Supplemental Tables

Table S1: Basic information about study participants

Group	Abbreviation	Gender	Age, years	Weight, kg	Height, cm	Regular smoker
oral	O1	female	26	88	174	no
	O2	male	30	71	182	no
	O3	female	29	58	159	no
inhalative	I1	female	29	80	172	no
	I2	male	25	90	195	yes
	I3	male	27	69	184	yes

Table S2: Sample collection schedule for serum, saliva, and urine following inhalative or oral consumption

	**Inhalative**		**Oral**	
	**Serum**	**Urine**	**Serum**	**Urine**
	Before consumption		Before consumption	
	After first inhalation		After swallow	
Minutes	3			
	6			
	9		10	
	12			
	15			
	18			
	21		20	
	24			
	27			
	30	30	30	
	35			
	40		40	
	45			
	50		50	
	55			
Hours	1	1	1	1
	1.25		1.25	
	1.5		1.5	
	1.75		1.75	
	2	2	2	2
			2.25	
	2.5		2.5	
			2.75	
	3	3	3	3
	4	4	4	4
	5		5	
	6	6	6	6
	7		7	
	8	8	8	8
		10		10
		12		12
	24	24	24	24
	48	48	48	48
		72		72
		96		96
		120		120
